# Exploring the Different Degrees of Magnetic Disorder in Tb_*x*_R_1−*x*_Cu_2_ Nanoparticle Alloys

**DOI:** 10.3390/nano10112148

**Published:** 2020-10-28

**Authors:** Elizabeth M. Jefremovas, María de la Fuente Rodríguez, Javier Alonso, Jesús Rodríguez Fernández, José Ignacio Espeso, Inés Puente-Orench, Daniel P. Rojas, Ana García-Prieto, María Luisa Fdez-Gubieda, Lidia Rodríguez Fernández, Luis Fernández Barquín

**Affiliations:** 1 Departamento CITIMAC, Facultad de Ciencias, Universidad de Cantabria, 39005 Santander, Spain; maria.delafuente@unican.es (M.d.l.F.R.); javier.alonsomasa@unican.es (J.A.); jesus.rodriguez@unican.es (J.R.F.); jose.espeso@unican.es (J.I.E.); barquinl@unican.es (L.F.B.); 2 Institut Laue-Langevin, 71 Avenue des Martyrs, CS 20156, CEDEX 9, 38042 Grenoble, France; puenteorench@ill.fr; 3 Instituto de Ciencia de Materiales de Aragón, CSIC, Pedro Cerbuna 12, 50009 Zaragoza, Spain; 4 Departamento Estructuras y Física de la Edificación, ETSAM, Universidad Politécnica de Madrid, 28040 Madrid, Spain; d.rojas@upm.es; 5 Departamento de Física Aplicada, Escuela de Ingeniería de Bilbao, 48013 Bilbao, Spain; ana.garciap@ehu.eus; 6 Departamento de Electricidad y Electrónica, Universidad del País Vasco—UPV/EHU, 48940 Leioa, Spain; malu.gubieda@ehu.eus; 7 SERMET-SCTI, Universidad de Cantabria, 39005 Santander, Spain; lidia.rodriguezf@unican.es

**Keywords:** magnetic nanoparticles, nanomagnetism, magnetic coupling, neutron diffraction, spin glass

## Abstract

Recently, potential technological interest has been revealed for the production of magnetocaloric alloys using Rare-Earth intermetallics. In this work, three series of Tb${}_{x}$R${}_{1-x}$Cu${}_{2}$ (R ≡ Gd, La, Y) alloys have been produced in bulk and nanoparticle sizes via arc melting and high energy ball milling. Rietveld refinements of the X-ray and Neutron diffraction patterns indicate that the crystalline structure in all alloys is consistent with TbCu${}_{2}$ orthorhombic Imma bulk crystalline structure. The analyses of the DC-magnetisation ($M_{DC}$) and AC-susceptibility ($\chi_{AC}$) show that three distinct degrees of disorder have been achieved by the combination of both the Tb${}^{3+}$ replacement (dilution) and the nanoscaling. These disordered states are characterised by transitions which are evident to $M_{DC}$, $\chi_{AC}$ and specific heat. There exists an evolution from the most ordered Superantiferromagnetic arrangement of the Tb${}_{0.5}$La${}_{0.5}$Cu${}_{2}$ NPs with Néel temperature, $T_{N}∼$ 27 K, and freezing temperature, $T_{f}∼$ 7 K, to the less ordered weakly interacting Superparamagnetism of the Tb${}_{0.1}$Y${}_{0.9}$Cu${}_{2}$ nanoparticles ($T_{N}$ absent, and T${}_{B}∼$ 3 K). The Super Spin Glass Tb${}_{0.5}$Gd${}_{0.5}$Cu${}_{2}$ nanoparticles ($T_{N}$ absent, and $T_{f}∼$ 20 K) are considered an intermediate disposition in between those two extremes, according to their enhanced random-bond contribution to frustration.

## 1. Introduction

Canonical Spin Glasses (SG) have been traditionally formed by doping weakly noble metals with magnetic ions coming from 3d transition metals [1,2]. In these kinds of systems, both frustration and disorder are achieved thanks to the random substitution (dilution) of the non-magnetic ions. Over the last few decades, the traditional noble metals have been replaced by magnetic Rare Earth (R) (e.g., RMn${}_{2}$ [3]), opening up the possibility for the occurrence of more complex magnetic phenomena. These have been especially attractive for spintronics or magneto-optical recording applications (e.g., RCo${}_{12}$B${}_{6}$ [4]). The situation becomes even more interesting when the 3d ion is non-magnetic (as in Cu [5] or Al [6]), as the starting magnetic ordered state can be tuned following two routes: on the one hand, one can modify the random-bond contribution by using 4f ions as dopants [7,8]. On the other hand, it has been showed that reducing the alloy size to the nanoscale affects the RKKY interactions among the magnetic moments, owing to both finite size effects [9,10,11] and the microstrain associated with grain boundaries [12,13,14]. These usually result in a combination of both frustration and random-site disorder that usually lead to the onset of a SG phase [15,16]. The question here that remains open is what happens to the disorder if one combines both worlds: dilution using 4f dopant ions plus size reduction to the nanoscale. Under these considerations, the present study explores the different degrees of magnetic disorder in three series of Tb${}_{x}$R${}_{1-x}$Cu${}_{2}$ alloys by tuning the strength of the RKKY interactions by combining both the magnetic dilution and the size reduction. These two ingredients act as a switch to turn on/off the different degrees of magnetic disorder in crystalline ordered structures.

Furthermore, this capability to control the magnetic moment orientation in frustrated magnets by tuning the intraparticle interactions may lead to complex magnetic arrangements, and it is the basic ingredient to understand the importance of emerging applications. In this way, these kinds of alloys are especially interesting for research on magnetic skyrmions using NP ensembles, which are promising candidates for future spintronic devices [17,18]. In addition, a recent work on R${}_{2}$RhSi${}_{3}$ compounds, where R ≡ Gd, Tb, and Dy [19] have opened the door to the 4f Gd and Tb ions to be used as potential candidates to obtain exotic magnetic materials. Furthermore, Gd-based compounds have been reported to display Giant Magnetocaloric effect (MCE), as in the case of Gd${}_{5}$(Si${}_{2}$Ge${}_{2}$) [20]. A recent study [21] has evidenced the RCu${}_{2}$ family to be potential candidates for low temperature refrigeration applications due to their large MCE at T<70 K. As the size reduction is expected to enhance the MCE [22], these RCu${}_{2}$ alloys could be considered potential candidates for the cooling technique in Nano Electro Mechanical Systems [23]. Within this framework, it is clear that a good understanding of the magnetic intraparticle interactions in these magnetically disordered alloys becomes mandatory [24,25].

For this purpose, we have produced three series of diluted bulk and NPs alloys, using the antiferromagnetic (AF) TbCu${}_{2}$ bulk alloy as a starting point. We have selected Gd${}^{3+}$, La${}^{3+}$ and Y${}^{3+}$ as diluting ions, producing Tb${}_{0.5}$Gd${}_{0.5}$Cu${}_{2}$, Tb${}_{0.5}$La${}_{0.5}$Cu${}_{2}$ and Tb${}_{0.1}$Y${}_{0.9}$Cu${}_{2}$ series of bulk, *t* = 2 h and 5 h milled NP alloys. Whereas La${}^{3+}$ and Y${}^{3+}$ are non-magnetic, Gd${}^{3+}$ displays, after Tb${}^{3+}$, the highest magnetic orbital moment *J* among the Lanthanides. This combination of two different magnetic ions is expected to enhance the random-bond disorder. In this work, we have observed that all the alloys retain the orthorhombic Imma crystalline structure showed by the non-diluted parent alloys (i.e., TbCu${}_{2}$ [16], GdCu${}_{2}$ [26]). Nevertheless, the magnetic behavior of the diluted alloys is clearly different from the one of the parents alloys already at the bulk state. In this way, this work shows that the random-bond disorder is enhanced in the whole Tb${}_{0.5}$Gd${}_{0.5}$Cu${}_{2}$ series. On the other hand, an increase of the random-site disorder is evidenced in the series of alloys with non-magnetic ions, Tb${}_{0.5}$La${}_{0.5}$Cu${}_{2}$, and Tb${}_{0.1}$Y${}_{0.9}$Cu${}_{2}$. This disorder has been achieved by altering the effective distance among the Tb${}^{3+}$, as some of the lattice positions are occupied by La${}^{3+}$ or Y${}^{3+}$, whose atomic radii differ more strenuously from the one of Tb${}^{3+}$ [27]. An evolution to different *zoologies* of disorder has been observed in the three series of alloys with the size reduction, opening the path to tune the RKKY interactions among the magnetic moments by controlling the NP size and the alloy composition. A good understanding of the different degrees of disorder is essential to tune the relevant parameters of these alloys in different applications, for example, as MCE agents [21].

## 2. Experimental Details

Polycrystalline pellets of Tb${}_{x}$R${}_{1-x}$Cu${}_{2}$ have been obtained in an arc furnace (MAM-1, Johanna Otto Gmbh, Germany) under an Ar atmosphere (99.99%) using the appropriate stoichiometric amounts of Gd, Tb, Y, and Cu pure metals. In the case of Gd and La, *x* = 0.5, while for Y, *x* = 0.1. The resulting powder was sealed-off under Ar pressure (99.99%) to avoid the oxidation, and grinded for 2 h and 5 h in a high-energy planetary ball mill (Retsch PM 400/2, Germany) in order to achieve nanometric sizes. The temperature was kept constant (50–60 ${}^{\circ }$C) during the whole milling process.

The structural characterisation has been performed by employing three advanced techniques [28]: X-ray Diffraction (XRD), Transmission Electron Microscopy (TEM), and Neutron Diffraction (ND). XRD measurements were performed in all alloys at room temperature in a Bruker D8 Advance diffractometer (Germany), using Cu-Kα (λ = 1.5418 Å) radiation. TEM measurements were performed in Tb${}_{0.5}$Gd${}_{0.5}$Cu${}_{2}$ NPs using a Jeol 2100 Microscope (Japan) (0.23 nm point resolution) equipped with an Oxford Inca X-stream EDX spectrometer (Japan). ND patterns were collected at D1B instrument (Institute Laue-Langevin, ILL, France) using a wavelength λ = 2.520 Å for bulk and 2 h milled Tb${}_{0.5}$La${}_{0.5}$Cu${}_{2}$ alloys at temperatures between T= 5 K and 300 K. Each of the patterns was measured for 8 h in order to get a high signal/noise ratio.

The magnetic characterisation (static M${}_{DC}$ and dynamic $\chi_{AC}$) was performed in both Quantum Design QD-PPMS and QD-MPMS (SQUID) magnetometers (CA, USA) (*T* = 2–300 K, $\mu_{0}H$ ≤ 9 T). For the $\chi_{AC}$, an oscillating field $\mu_{0}H$ = 0.313 mT and frequencies (*f*) ranging from 0.01 kHz to 10 kHz were employed. Additionally, bulk Tb${}_{0.1}$Y${}_{0.9}$Cu${}_{2}$ alloy has been measured in the lower frequency range of *f* = 0.3–300 Hz with $\mu_{0}H$ = 0.1 mT.

Heat capacity (c${}_{p}$) measurements were performed in Tb${}_{0.5}$Gd${}_{0.5}$Cu${}_{2}$ (bulk and 2 h-milled NPs) and in Tb${}_{0.5}$La${}_{0.5}$Cu${}_{2}$ (bulk, 2 h, and 5 h) using the QD-PPMS instrument (*T* = 2–300 K, $\mu_{0}H$ ≤ 8 T) following the relaxation method [29].

## 3. Results and Discussion

### 3.1. Structural Characterisation: XRD

**Figure 1:**
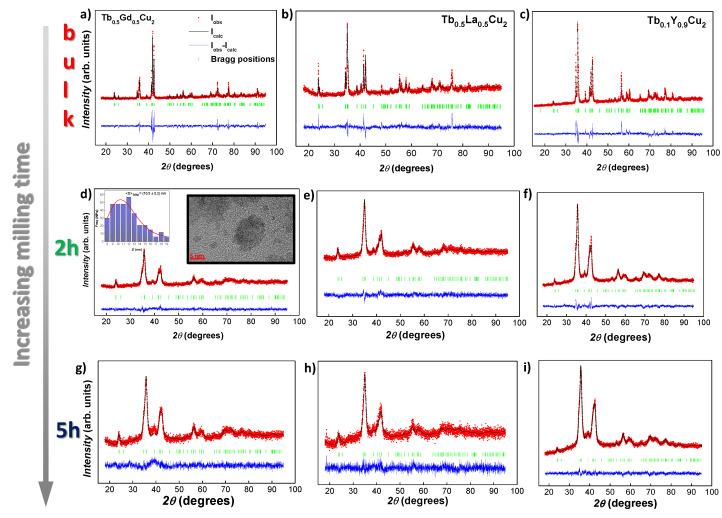
XRD patterns for Tb${}_{0.5}$Gd${}_{0.5}$Cu${}_{2}$ bulk (**a**), 2 h (**d**) and 5 h milled NPs (**g**), Tb${}_{0.5}$La${}_{0.5}$Cu${}_{2}$ bulk (**b**), 2 h (**e**) and 5 h milled NPs (**h**) and Tb${}_{0.1}$Y${}_{0.9}$Cu${}_{2}$ bulk (**c**), 2 h (**f**) and 5 h milled NPs (**i**). Experimental data are shown in red, theoretical calculation (Rietveld refinement) in black, and the difference between the calculated and experimental patterns is shown by the blue line below the spectrum. Additionally, the inset of (**d**) shows a TEM image for the Tb${}_{0.5}$Gd${}_{0.5}$Cu${}_{2}$-2 h milled NPs together with the size distribution fitted to a LogNormal distribution.

Figure 1 includes the XRD patterns with the performed Rietveld refinements corresponding to the three series of the produced alloys. All patterns are consistent with a single crystallographic phase of the orthorhombic CeCu${}_{2}$-type crystal structure (Imma space group), as it is found in the parent bulk RCu${}_{2}$ alloys (R≡ Tb, Gd or Y). The R${}^{3+}$ ions occupy the *4e*-sites (0, 0.25, z), whereas Cu atoms are located at the *8h* position (0, x, y). Values for *x*, *y*, and *z* are found to lie near *x*≈ 0.006, *y* ≈ 0.163, and *z* ≈ 0.547. However, LaCu${}_{2}$ is an exception for this orthorhombic Imma structure, as it crystallises in a hexagonal P6/mmm AlB${}_{2}$-type one [30]. This implies that the crystalline structure of the Tb${}_{0.5}$La${}_{0.5}$Cu${}_{2}$ alloy could consist of a mixture of both orthorhombic and hexagonal phases. Nevertheless, the Rietveld refinements [shown in Figure 1b,e,h] reveal unambiguously that only a single phase of the orthorhombic Imma structure is present. This fact is in agreement with the lower energy-cost of an orthorhombic structure with respect to the hexagonal AB${}_{2}$-type [5].

The main structural parameters for the bulk and NP alloys are summarised in Table 1. First of all, the Bragg error factors $R_{B}$ are kept below 10%, which ensures the reliability of our refinements. The lattice parameters of the bulk diluted alloys are slightly decreased with respect to the ones of the bulk parent TbCu${}_{2}$ and GdCu${}_{2}$ [5], leading to a small reduction of the unit cell volume. This general trend is in good agreement with the one previously observed in a Gd${}_{x}$Y${}_{1-x}$Cu${}_{2}$ bulk alloy [7]. Nevertheless, an exception for this trend is found in Tb${}_{0.5}$La${}_{0.5}$Cu${}_{2}$, where the unit cell is expanded with respect to the TbCu${}_{2}$ bulk alloy. The greater ionic radii of La${}^{3+}$ ions (r=1.032 Å [27]) in comparison to to Tb${}^{3+}$ (r=0.923 Å [27]) could be the reason for this.

Figure 1d–i displays the XRD patterns for the nanoscaled alloys (*t* = 2 h and *t* = 5 h, respectively). According to the Rietveld refinements, the orthorhombic Imma crystalline structure is maintained. As it can be observed from the values included in Table 1, the unit cell tends to expand when the bulk powders are milled for the La${}^{3+}$ and Y${}^{3+}$ alloys, whereas the dilution with Gd${}^{3+}$ experiences a unit cell contraction. This effect can be attributed to the different metallurgical behaviour of the alloys [31,32].

Regarding the NPs’ mean size, it can be seen, according to Table 1, that Tb${}_{0.5}$Gd${}_{0.5}$Cu${}_{2}$ and Tb${}_{0.1}$Y${}_{0.9}$Cu${}_{2}$ reach a mean diameter size 〈D〉∼ 10 nm after milling for *t* = 2 h, and 〈D〉∼ 7 nm after *t* = 5 h. Nevertheless, Tb${}_{0.5}$La${}_{0.5}$Cu${}_{2}$ NPs display greater sizes and smaller microstrain values. This may suggest that including La${}^{3+}$ ions could favour a harder metallurgical resistance to the grinding. All of the produced alloys display microstrain values below ∼1%, which ensures their good crystallinity.

Finally, a TEM image for Tb${}_{0.5}$Gd${}_{0.5}$Cu${}_{2}$-2 h milled NPs is shown in the inset of Figure 1d. This technique has been employed to check the crystalline microscopic structure of the Tb${}_{0.5}$Gd${}_{0.5}$Cu${}_{2}$ NPs, as no ND measurements could be performed for this dilution due to the high absorption rate of Gd [33]. The clearly depicted crystallographic planes confirm the crystallinity of the NPs. Furthermore, the size-distribution (inset) reveals the usually found LogNormal distribution for mean NP sizes, with a mean size diameter of $D_{TEM}$ = 10.5 (2) nm. This result is in good agreement with the 〈D〉= 9.0(8) nm obtained from the Rietveld refinements of the XRD patterns.

### 3.2. Structural Characterisation: Neutron Diffraction

**Figure 2:**
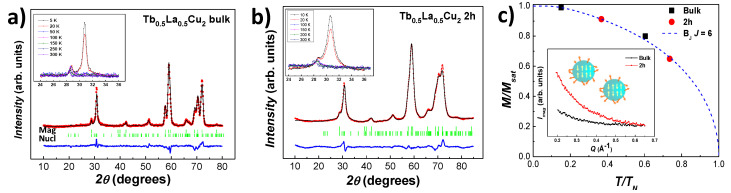
Tb${}_{0.5}$La${}_{0.5}$Cu${}_{2}$ ND patterns measured with λ=2.520 Å for (**a**) bulk (T= 5 K) and (**b**) 2 h milled NPs (T= 10 K). The Rietveld refinements (black) agree with the experimental data (red), as it can be observed from the difference I${}_{obs}$-I${}_{calculated}$ (blue line). The nuclear and magnetic Bragg reflections are shown in green. The insets show the emergence of the magnetic peak located at 2θ∼ 30${}^{\circ }$ when the temperature is decreased; (**c**) evolution of the magnetisation per Tb${}^{3+}$ atom (M/M${}_{sat}$) with the temperature for bulk (black) and 2 h milled NPs (red). The blue-dotted line represents the Brillouin function calculated with *J* = 6. The inset shows the variation of the magnetic intensity in the low *Q* region (Q<0.665 Å${}^{-1}$) for bulk (black) and 2 h NPs (red) at T= 20 K.

Microscopic magnetic structure analyses were performed on Tb${}_{0.5}$La${}_{0.5}$Cu${}_{2}$ bulk and nano (*t* = 2 h) alloys. Figure 2a shows the Neutron Diffraction (ND) pattern for the bulk alloy measured at *T* = 5 K (magnetic state). Experimental data have been fitted by employing a Rietveld refinement for both the magnetic and the nuclear structures. The achieved low Bragg factors (R${}_{B}^{mag}$ = 10.6 % and R${}_{B}^{nuclear}$ = 8.1%) guarantee the reliability of the fits. The appearance of the magnetic structure is clearly observable for T≤ 20 K (see inset), as two clear magnetic peaks within the range 28${}^{\circ }$<2θ< 33${}^{\circ }$ show up. This finding is in good agreement with the AF state that takes place at $T_{N}=33.1\left(1\right)$ K (see magnetic characterisation below). The magnetic structure has been determined to be collinear commensurate AF with two propagation vectors $\tau_{1}$ = (0, 0, 0) and $\tau_{2}$ = (1/3, 0, 0), where the magnetic moments are aligned along the a-axis direction. The thermal evolution of the magnetisation per Tb${}^{3+}$ atom (M/M${}_{sat}$) (see Figure 2c black dots) follows a Brillouin dependency with J= 6. The saturation value is 20% decreased with respect to the bulk TbCu${}_{2}$ [16], which can be attributed to the reduced coordination of Tb${}^{3+}$ plus the disorder associated with the La${}^{3+}$ substitution. For this Tb${}_{0.5}$La${}_{0.5}$Cu${}_{2}$ bulk alloy, as for TbCu${}_{2}$ [34], the magnetic moments show two different temperature dependencies, depending on their Miller index: the ones indexed with odd Miller index (*h*, *k*, *l*) decrease faster, when the temperature increases, than the ones indexed with even *h* + *k* + *l* and (h±1/3, *k*, *l*).

Figure 2b shows the ND pattern measured for 2 h milled Tb${}_{0.5}$La${}_{0.5}$Cu${}_{2}$ NPs at *T* = 10 K (magnetic state), where a nuclear $R_{B}$ = 7 % and magnetic $R_{B}$ = 10% have been achieved. The presence of a well-defined magnetic structure at *T*≤20 K that gets more visible when lowering the temperature (see inset) is a direct revelation that the AF ordering survives within the NPs, as the lack of translation invariance of a SG state would prevent the magnetic Bragg peaks from appearing [1]. The magnetic characterisation (see below) will support this finding, as a Néel transition located at $T_{N}=$ 27.1(1) K is observed. The magnetic size obtained from the Rietveld refinements reveals a single-domain ensemble of nanoparticles, as $\langle D_{mag}\rangle$ = 12.3(3) nm, which is close to the nuclear $\langle D_{nucl}\rangle$ = 13.8(4) nm. The obtained value for $\langle D_{nucl}\rangle$ is in good agreement with the NP size obtained by means of XRD measurements (〈D〉 = 12.9(8) nm). The unit cell parameters (not shown) tend to shrink when lowering the temperature, as for the bulk alloy. Here again, the magnetic moments indexed with odd (*h*, *k*, *l*) decrease faster with the temperature, as for the bulk state. A Brillouin-like dependency for (M/M${}_{sat}$) with J= 6 is recovered for this case too (see Figure 2c red dots).

Finally, the inset of Figure 2c shows the low-Q region (2${}^{\circ }$< 2θ< 15${}^{\circ }$, i.e., Q<0.665 Å${}^{-1}$) for both the bulk and the 2 h-milled NPs measured at *T* = 20 K. The magnetic signal increases for the nanoscaled alloy, pointing to the existence of interparticle correlations. The provenance of these correlations is related to the increasing disorder of the magnetic moments, driven by both the size reduction and the microstrain. Such correlations bring to light the interacting Spin Glass (SG) nature of those disordered magnetic moments (located at the shell), rather than a non-interacting SPM arrangement. Later on, the magnetic characterisation will support this finding.

All in all, we can successfully determine the magnetic state for these Tb${}_{0.5}$La${}_{0.5}$Cu${}_{2}$ NPs as a Super Antiferromagnetic (SAF) [see simple sketch included in Figure 2c], where the magnetic moments located within the core are AF ordered while the ones at the shell are in a disordered Spin Glass state. This also happened for the parent TbCu${}_{2}$ NPs [16]. The magnetic characterisation shown hereunder will further support this finding.

### 3.3. Magnetic Characterisation

#### 3.3.1. Static Magnetic Susceptibility

**Figure 3:**
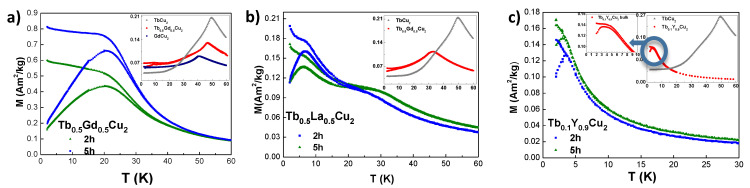
ZFC-FC magnetisation t= 2 h (blue squares) and 5 h (green triangles) milled NPs collected at $\mu_{0}H$ = 10 mT for: (**a**) Tb${}_{0.5}$Gd${}_{0.5}$Cu${}_{2}$; (**b**) Tb${}_{0.5}$La${}_{0.5}$Cu${}_{2}$ and (**c**) Tb${}_{0.1}$Y${}_{0.9}$Cu${}_{2}$. Insets show the bulk diluted alloy (red circles) with respect to the non diluted parents GdCu${}_{2}$ (dark blue squares) and/or TbCu${}_{2}$ (gray triangles). The *y*-axis has been re-scaled in all of the alloys for clarity purposes.

Figure 3a–c shows the Zero-Field Cooled (ZFC) and Field Cooled (FC) magnetisation $M_{DC}\left(T\right)$ measurements performed at low field ($\mu_{0}H$ = 10 mT) for the three series of alloys. The temperature values corresponding to the observed transitions and the values obtained from a Curie–Weiss fitting performed on the data measured at a $\mu_{0}H=$ 10 mT (see Figure S1 in Supplementary Materials) can be inspected in Table 2.

We will start by discussing the analyses concerning the Tb${}_{0.5}$Gd${}_{0.5}$Cu${}_{2}$ series. As it can be seen from Figure 3a, the ZFC and FC branches for the NPs display no trace of AF Néel transition, which is however present in the bulk state (see inset). The bulk $T_{N}=$ 47.2(1) K lies between those corresponding to bulk GdCu${}_{2}$ ($T_{N}=$ 40.2(1) K [15]) and TbCu${}_{2}$ ($T_{N}$ = 49.1(1) K [35]) alloys. The magnetisation value at this transition also lies between the parents’ ones (almost 2.25 times larger than the one of GdCu${}_{2}$ and 1/3 of the value of TbCu${}_{2}$). It is worth noting the occurrence of an irreversibility already for the bulk state at T≲ 18 K, which is a hint of the existence of a Spin Glass state. This disordered magnetic state would be triggered by the random-bond disorder plus the competition between AF and FM interactions. The latter is revealed by the positive value of the paramagnetic Curie temperature $\theta_{P}\approx 20$ K (see Table 2). The presence of a disordered magnetic phase already at the bulk state has also been shown in other Gd intermetallics, such as in pollycrystalline Gd${}_{4}$PtAl [36] or GdCu${}_{2}$ [15], where the obtained $\theta_{P}\approx 20$ K agrees well with the one obtained in our Tb${}_{0.5}$Gd${}_{0.5}$Cu${}_{2}$ bulk. For the NPs, a clear freezing transition $T_{f}$ takes place (at around 20 K), leading to the formation of a Super Spin Glass state (SSG) [37]. This evolution from a bulk AF state to a SSG for the NPs has already been shown in other systems, as in GdCu${}_{2}$ [15] or in the 3d NiO compounds [38]. The SSG state gets more robust for smaller NP sizes [37,39], denoted by an increase for both magnetisation (1.5 times) and freezing transition $T_{f}$ ($\frac{T_{f}^{7nm}-T_{f}^{9nm}}{T_{f}^{9nm}}\approx$ 8% ) when comparing alloys *t* = 2 and 5 h. The value of $\theta_{P}$ is still positive for the NPs, but shows a smooth reduction with size. Such a finding is concomitant with a progressive weakening of the FM interactions due to the increasing number of shell magnetic moments (disordered). Finally, the obtained $\mu_{eff}$ values do not display appreciable size-dependence, and lie slightly below the ones reported for parent TbCu${}_{2}$ and GdCu${}_{2}$ [15,16].

We will now discuss the results obtained for the Tb${}_{0.5}$La${}_{0.5}$Cu${}_{2}$ series. Here, opposite to what happened in Tb${}_{0.5}$Gd${}_{0.5}$Cu${}_{2}$, an AF transition that takes place at $T_{N}\approx 33$ K, is kept in both bulk and NP state, as it can be seen from the ZFC-FC measurements represented in Figure 3b. Additionally to this Néel transition, the NPs do also experience a freezing process that takes place at $T_{f}∼$ 6–7 K. Thereby, a Superantiferromagnetic state (SAF) [35,37]) should be considered, for which the core magnetic moments are AF coupled while the shell ones are forming a SG state, in good agreement with the ND measurements shown in Section 3.2. The results reveal that, although the magnetisation at the AF transition is almost constant for bulk and the NPs, the $T_{N}$ values are slightly decreased (see Table 2). Accordingly, a reduction of $\left|\Delta T_{N}\left(9nm\right)\right|=\frac{T_{N}\left(9nm\right)-T_{N}\left(bulk\right)}{T_{N}\left(bulk\right)}\approx 20\%$ is quantified, which is almost twice the reduction that was obtained in the case of pure TbCu${}_{2}$ NPs [16]. This is in clear agreement with the fact that the Tb${}^{3+}$-content has been reduced to 50% in the diluted alloy. A reduction of the magnetisation value at the AF transition to half of the one corresponding to TbCu${}_{2}$ is found (see inset of Figure 3b), which has also been observed in Tb${}_{0.5}$Y${}_{0.5}$Cu${}_{2}$ single-crystal [40]. These results support the claim that the weakening of the RKKY exchange interactions in these alloys is solely affected by the replacement of Tb${}^{3+}$ ions by non-magnetic R${}^{3+}$ ones, regardless of the particular element. Memory effects revealing frustration appear already for the bulk sample for T≲ 20 K, triggered once again by competing FM-AF interactions. The obtained $\mu_{eff}$ values do not display appreciable size-dependence and lie slightly below the ones reported for parent TbCu${}_{2}$ [16].

We will finish by discussing the measurements of the Tb${}_{0.1}$Y${}_{0.9}$Cu${}_{2}$ series, which can be found in Figure 3c. Here, differently from the case of bulk Tb${}_{0.5}$Gd${}_{0.5}$Cu${}_{2}$ or Tb${}_{0.5}$La${}_{0.5}$Cu${}_{2}$ alloys, no trace of the Néel transition is found either in the bulk or in the NP states. This is coherent with the lack of both metamagnetism and hysteresis reported for this alloy [8,41]. Particularly, in [41], a critical value of x${}_{c}$ = 0.15 was stated as the minimum concentration of Tb${}^{3+}$-ions needed to give rise to a global AF state. Nevertheless, even if the interactions are weakened, the magnetic moments do interact among them, which is evidenced by the irreversibility found in the form of a plateau in the FC branch at low temperatures (see central inset). This irreversibility can be associated with a Cluster Spin Glass state (CSG), as will be revealed by the dynamic $\chi_{AC}$ measurements described below. The value of the $T_{f}$ is reduced in the NPs, pointing to weaker interactions among the magnetic moments, which is contrary to what was described above for Tb${}_{0.5}$Gd${}_{0.5}$Cu${}_{2}$ and Tb${}_{0.5}$La${}_{0.5}$Cu${}_{2}$. Bearing in mind that only 10 % of the moments are magnetic in the Tb${}_{0.1}$Y${}_{0.9}$Cu${}_{2}$ alloy, the reduction of the total amount of the Tb${}^{3+}$ contained in each NP (as a result of the size reduction) reduces the strength of the competing FM-AF RKKY interactions. This yields to a less interacting ensemble of magnetic moments, resulting in a reduction of the random-bond contribution to frustration, thus a weaker SG state. This progressive weakening leaves also a trace in the FC branch, as the expected *plateau*-shape for $T<T_{f}$ is absent, which can only mean that the magnetic NPs relax more independently [1]. A de Almeida–Thouless analysis [42,43] (shown in Figure S1 in Supplementary Materials) of the freezing temperature with the applied field according has been performed to check out the nature of this SG state in these alloys. The analysis indicates that only the bulk alloy presented an SG-like behaviour, with *m* = 3/2. Furthermore, 5 h-milled NPs display a behaviour more similar to the one characteristic of a Superparamagnetic (SPM) ensemble of NPs, where a blocking temperature $T_{B}$ must be considered rather than a freezing temperature. The rise in the FC branch for $T<T_{B}$ supports the evolution from a CSG in the bulk state to a weak interacting SPM state in 5 h-milled NPs. In this series of Tb${}_{0.1}$Y${}_{0.9}$Cu${}_{2}$ alloys, $\mu_{eff}$ values are again close to the experimental value of TbCu${}_{2}$ [16].

#### 3.3.2. Isothermal Magnetisation

**Figure 4:**
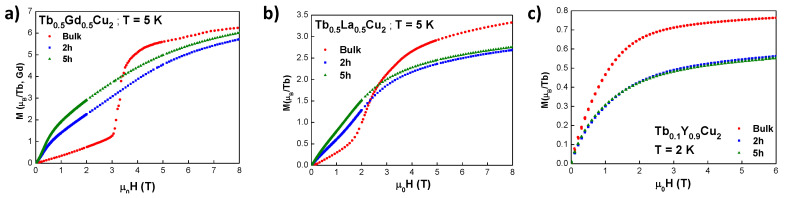
*M* vs. $\mu_{0}H$ curves for bulk (red circles), 2 h (blue squares) and 5 h (green triangles) milled NPs measured at *T* = 5 K for (**a**) Tb${}_{0.5}$Gd${}_{0.5}$Cu${}_{2}$ and (**b**) Tb${}_{0.5}$La${}_{0.5}$Cu${}_{2}$, and *T* = 2 K for (**c**) Tb${}_{0.1}$Y${}_{0.9}$Cu${}_{2}$ alloys.

Isothermal $M_{DC}\left(\mu_{0}H,T\right)$ measurements of the diluted alloys are shown in Figure 4. A temperature of T= 5 K was employed for both Tb${}_{0.5}$Gd${}_{0.5}$Cu${}_{2}$ and Tb${}_{0.5}$La${}_{0.5}$Cu${}_{2}$, whereas a T= 2 K was needed for the Tb${}_{0.1}$Y${}_{0.9}$Cu${}_{2}$ dilution, as the SG phase appeared at T≤ 4 K. First, it is worth mentioning the metamagnetic transitions located at $\mu_{0}H$ = 3.33(1) T for Tb${}_{0.5}$Gd${}_{0.5}$Cu${}_{2}$ (Figure 4a) and $\mu_{0}H$ = 2.31(1) T for Tb${}_{0.5}$La${}_{0.5}$Cu${}_{2}$ (Figure 4b). Whereas the shape of the metamagnetic transition of Tb${}_{0.5}$La${}_{0.5}$Cu${}_{2}$ is more similar to a spin-flop mechanism, the one for Tb${}_{0.5}$Gd${}_{0.5}$Cu${}_{2}$ is spin-flip like, pointing to a higher anisotropy for the latter alloy [44]. No hint of this transition is found for bulk Tb${}_{0.1}$Y${}_{0.9}$Cu${}_{2}$ (Figure 4c), according to its CSG state. This Tb${}_{0.1}$Y${}_{0.9}$Cu${}_{2}$ bulk alloy also displays the smallest anisotropy value of the produced dilutions, as it is almost saturated at $\mu_{0}H$ = 4 T, while the magnetic saturation is not reached for Tb${}_{0.5}$Gd${}_{0.5}$Cu${}_{2}$ or Tb${}_{0.5}$La${}_{0.5}$Cu${}_{2}$ at $\mu_{0}H$ = 8 T (in the same way as the bulk parent alloys [5]). All the obtained $M(\mu_{0}H)$ values for each alloy agree well with their Tb${}^{3+}$ content. The value of M(6T) = 0.765(1) $\mu_{B}$/ Tb for the Tb${}_{0.1}$Y${}_{0.9}$Cu${}_{2}$ bulk alloy is almost 10 times decreased with respect to the TbCu${}_{2}$ value at same $\mu_{0}H$ [16], and the values found for M(8T) are ≈ 89% and ≈ 50% from the corresponding to TbCu${}_{2}$ measured at the same field [16] for Tb${}_{0.5}$Gd${}_{0.5}$Cu${}_{2}$ and Tb${}_{0.5}$La${}_{0.5}$Cu${}_{2}$, respectively.

**Figure 5:**
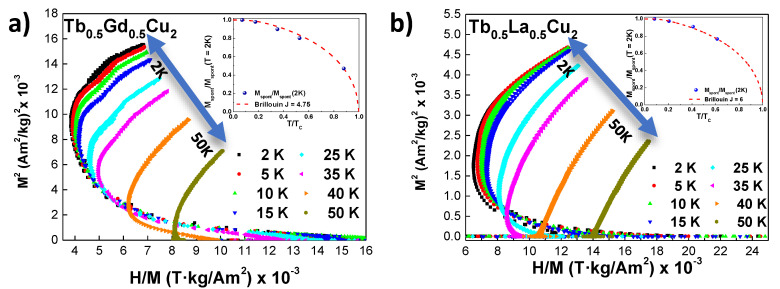
$M^{2}$ vs $\mu_{0}H/M$ Arrott plots for (**a**) Tb${}_{0.5}$Gd${}_{0.5}$Cu${}_{2}$ and (**b**) Tb${}_{0.5}$La${}_{0.5}$Cu${}_{2}$ bulk alloys. The insets represent the relative spontaneous magnetisation $M_{spont}/M_{spont}\left(2K\right)$ obtained from the Arrott plots in a relative temperature scale respect $T_{C}$. The dashed red line represent a Brillouin curve with *J* = 4.75 and *J* = 6 respectively. Values of $M_{spont}$ obtained for $T>T_{C}$ are equal to zero.

In order to elucidate some subtleties about the magnetic coupling to a external field, we have analysed the Arrott plots for both Tb${}_{0.5}$Gd${}_{0.5}$Cu${}_{2}$ and Tb${}_{0.5}$La${}_{0.5}$Cu${}_{2}$ AF bulk alloys at several temperatures below $T_{N}$. These Arrott plots are represented in Figure 5. First, we observe the expected lineal shape for an AF ordered state [45]. Nevertheless, we have found non-negligible values of spontaneous magnetisation ($M_{spont}$), which corroborate the existence of the incipient FM interactions already discussed in the $M_{DC}\left(T\right)$ section. The evolution with the temperature for these $M_{spont}$ follows a Brillouin-like function (see insets) [45], as expected for FM. The obtained FM parameters for Tb${}_{0.5}$Gd${}_{0.5}$Cu${}_{2}$ are $T_{C}$ = 28.5(1) K and *J* = 4.75, which agree with a proportion of 50 % of Tb${}^{3+}$ (J=6) and 50 % of Gd${}^{3+}$ (*J* = 7/2). This $T_{C}$ lies near the obtained $\theta_{P}$ value (see Table 2). For the Tb${}_{0.5}$La${}_{0.5}$Cu${}_{2}$ bulk alloy, the $T_{C}$ = 24.4(1) K (close to $\theta_{P}$) and *J* = 6 ($J_{Tb^{3+}}$ = 6). The fact of having competing AF and FM interactions gives rise to a magnetically disordered phase, which is evidenced by the finding of a right-curvature at low *M*${}^{2}$ values [46]. This curvature is visible at *T*≤ 25 K for Tb${}_{0.5}$Gd${}_{0.5}$Cu${}_{2}$ and *T*≤ 15 K for Tb${}_{0.5}$La${}_{0.5}$Cu${}_{2}$, in good agreement with the irreversibility observed in the FC branch.

If we analyse now the M(H) behaviour of the nanoscaled alloys, we can observe how the magnetisation gets reduced in the NP state. Both the canting of the magnetic surface moments and the increasing distance among the magnetic moments with respect to the bulk state can be addressed to understand this finding. Once in the NP regime, a further size reduction acts in favour of the magnetisation, as a slight increase can be noticed for t= 5 h NPs with respect to t= 2 h. This rise is explained by the growing anisotropy contribution coming from canted spins that increases as lattice microstrain and shell/core ratio do. In order to bring more light into the disordered state of the SPM Tb${}_{0.1}$Y${}_{0.9}$Cu${}_{2}$ 5 h milled NPs, hysteresis loops (not shown) have been performed at *T* = 2 K (i.e., below the blocking temperature), where non-negligible values for both coercive field ($\mu_{0}H_{C}$ = 8.82(1) mT) and remanence ($M_{r}$ = 14.4(1) Am${}^{2}$/kg) have been found. These findings reveal that the magnetic state is that of a weakly interacting SPM, rather than a pure non-interacting one [37]. An estimation of the dipolar interaction contribution gives a very reduced value of $E_{d-d}/k_{B}∼$ 0.22 K, which is far from the observed *T*${}_{B}\approx$ 3 K. This low E${}_{d}$ value prevents the dipolar interactions to develop a cooperative glassy state, as it was the case for bulk Tb${}_{0.1}$Y${}_{0.9}$Cu${}_{2}$.

#### 3.3.3. Dynamic Magnetic Susceptibility

**Figure 6:**
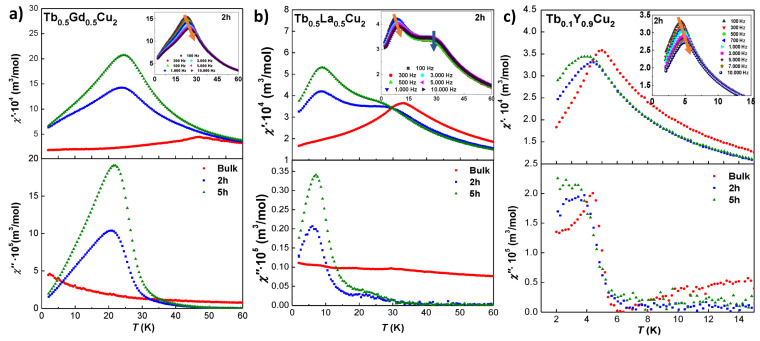
In-phase $\chi^{\prime }$(T) (top) and out-of-phase $\chi^{\prime \prime }$ (T) (bottom) for bulk (red circles), 2 h (blue squares) and 5 h (green triangles) milled NPs of (**a**) Tb${}_{0.5}$Gd${}_{0.5}$Cu${}_{2}$, (**b**) Tb${}_{0.5}$La${}_{0.5}$Cu${}_{2}$ and (**c**) Tb${}_{0.1}$Y${}_{0.9}$Cu${}_{2}$ measured at *f* = 1000 Hz [*f* = 100 Hz in (**c**)] and *h* = 0.313 mT. The inset shows the $\chi^{\prime }\left(T,f\right)$ evolution for 2 h milled NPs. The orange arrow indicates the freezing transition, whereas the blue one signals the invariant position for the Néel transition.

A detailed study on the magnetic dynamics becomes mandatory, as all alloys show magnetic irreversibility. Thereby, Figure 6a–c shows the behaviour of the in-phase [$\chi^{\prime }$ (T)] and out-of-phase [$\chi^{\prime \prime }$ (T)] components for bulk, 2 h, and 5 h milled alloys. As the qualitative results are coherent with the static M${}_{DC}$, we will just mention that the Néel transition is effectively absent for Tb${}_{0.1}$Y${}_{0.9}$Cu${}_{2}$ alloy and for the Tb${}_{0.5}$Gd${}_{0.5}$Cu${}_{2}$ NPs. Here, it is worth noting the rise of the [$\chi^{\prime \prime }$ (T)] signal already for Tb${}_{0.5}$Gd${}_{0.5}$Cu${}_{2}$ bulk at *T*< 20 K, which is connected to the conjectured existence of SG clusters. On the other hand, Tb${}_{0.5}$La${}_{0.5}$Cu${}_{2}$ NPs retain the AF transition. As it can be observed in the insets, the SG cusp follows the expected right-shift frequency dependence in all the alloys [6], whereas the Néel transition is frequency independent.

The frequency dependence of the freezing process has been first checked analysing the well known δ-parameter. By inspection of Table 3, it turns out that the alloys containing 50 % of Tb display δ-parameter values (0.05–0.08) larger than the ones expected for canonical Spin Glasses (δ≤0.04, [1]). These values are also higher than the ones reported for TbCu${}_{2}$ [16] or GdCu${}_{2}$ [15] NPs, but still below the ones for SPM systems with δ≥0.1 [37]. All in all, the obtained values lie close to those of CSG systems, δ∼0.06 [47]. In addition, a more consistent procedure is to analyse the validity of the critical slowing down law ${\left(\frac{T-T_{f,0}}{T_{f,0}}\right)}^{z\nu }$ followed in SG systems [1,48]. The obtained zν values are inside the *fragile regime* behaviour (5<z<11) [49]. The evolution with milling time does not show nearly any change for the Tb${}_{0.5}$La${}_{0.5}$Cu${}_{2}$ NPs. However, there is a clear decrease of the δ and zν values for Tb${}_{0.5}$Gd${}_{0.5}$Cu${}_{2}$ NPs, as expected for a more glassy state [50]. Values of T${}_{f\to 0}$ are slightly below the ones obtained for the freezing according to M${}_{DC}$ characterisation, which is expected, as the true phase transition is reached solely when H,f→ 0 [1]. In contrast, the evolution of the magnetic behaviour of the Tb${}_{0.1}$Y${}_{0.9}$Cu${}_{2}$ alloys is different, as an increase of δ, together with a reduction in both zν and T${}_{f,0}$, with milling time have been found. This implies weaker interactions for smaller NPs, as we have already argued. Following this idea, the values for δ support the transformation from a bulk CSG ensemble to a 5 h-milled NPs Superparamagnetic one. This change from a freezing process to a blocking mechanism explains that the fitting of the experimental $T_{B}$ for the Tb${}_{0.1}$Y${}_{0.9}$Cu${}_{2}$ 5 h according to a dynamic critical exponent fails, as no phase transition is established in this alloy.

**Figure 7:**
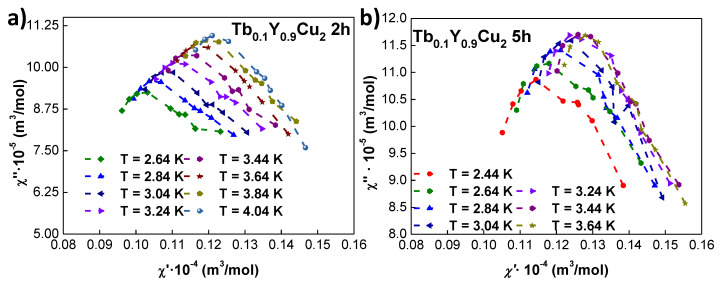
Cole–Cole diagrams for Tb${}_{0.1}$Y${}_{0.9}$Cu${}_{2}$ (**a**) 2 h and (**b**) 5 h milled NPs measured at several temperatures close below and above $T_{f}$ and ($T_{B}$).

To better understand the evolution in Tb${}_{0.1}$Y${}_{0.9}$Cu${}_{2}$ from the interacting SSG NPs to a weakly coupled SPM, we have represented in Figure 7 the Cole–Cole diagrams. These representations are a powerful tool to obtain information about the NP size distribution and anisotropy [51,52]. While the Cole–Cole diagram of an ideal monodisperse ensemble of SPM NPs should be a perfect semi-circle, our results on 5 h milled NPs [Figure 7a] are flattened and asymmetric semi-circles. This points to a polydisperse Log-normal particle size distribution [37]. The occurrence of a broad peak (maximum) in $\chi^{\prime \prime }\left(\chi^{\prime }\right)$ further supports this deviation from a monodisperse SPM ensemble of NPs. This maximum shows a right shift of $\chi^{\prime }\left(T=3.64\;K\right)-\chi^{\prime }\left(T=2.44\;K\right)∼0.017\times 10^{-4}$ m${}^{3}$/mol to higher $\chi^{\prime }$ values with increasing temperature. On the other hand, the Cole–Cole diagram of CSG 2 h milled represented in Figure 7b displays a more drastic right-shift of the maximum with increasing temperature, as $\chi^{\prime }\left(T=4.4\;K\right)-\chi^{\prime }\left(T=2.44\;K\right)∼0.029\times 10^{-4}$ m${}^{3}$/mol. This is to say, ≈ 1.7 times greater than the one for 5 h milled NPs. This is indicative of a narrower distribution of relaxation times, which is in good agreement with CSG state of 2 h milled NPs, i.e., a more interacting ensemble with respect to the SPM 5 h milled NPs. The fact that the $\chi^{\prime \prime }$ vs. $\chi^{\prime }$ curves show a flattened shifted downwards profile with respect to the situation for 5 h milled NPs further supports this more interacting nature, being a typical signature of frustrated cooperative interactions [1]. Finally, a relative breadth $\sigma_{rel}∼0.33$ can be calculated for this peak, which is clearly greater than the of $\sigma_{rel}∼0.05$ corresponding to an archetypal canonical spin glass of Au${}_{96}$Fe${}_{4}$ [53]. This finding further corroborates the cooperative cluster behaviour of the magnetic moments rather than the individual response of SG ones.

### 3.4. Specific Heat

Given the rich variety of magnetic order/disorder transitions observed in these alloys, specific heat $c_{p}$ measurements have been performed in Tb${}_{0.5}$Gd${}_{0.5}$Cu${}_{2}$ and Tb${}_{0.5}$La${}_{0.5}$Cu${}_{2}$ bulk and NPs to obtain more information about the nature of those transitions. No $c_{p}$ measurements have been performed in the Tb${}_{0.1}$Y${}_{0.9}$Cu${}_{2}$ series, as no evidence of order transitions is found.

The $c_{p}$ is then assumed to be the result of three contributions, following a standard analysis:

(1) $$c_{P}=c_{ph}+c_{e}+c_{mag}$$

**Figure 8:**
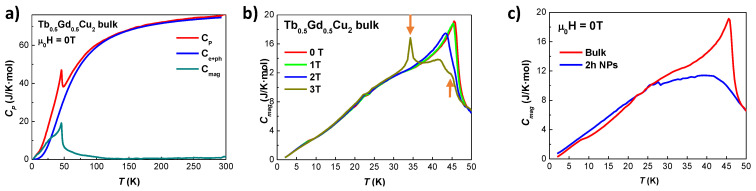
(**a**) Experimental specific heat $c_{P}$ for Tb${}_{0.5}$Gd${}_{0.5}$Cu${}_{2}$ bulk alloy vs. *T* measured at $\mu_{0}H$ = 0 T (red line), together with the c${}_{e+ph}$ contribution (blue line) and the $c_{mag}$ (green line); (**b**) $c_{mag}$ vs. *T* for the bulk alloy measured at $\mu_{0}H$ = 0 T (red line), 1 T (green), 2 T (blue) and 3 T (dark yellow). The dark orange arrows indicate the position for the two extra peaks. (**c**) Bulk (red line) and 2 h milled (nano) (blue) $c_{mag}$ contributions vs. *T* measured at $\mu_{0}H$ = 0 T.

**Figure 9:**
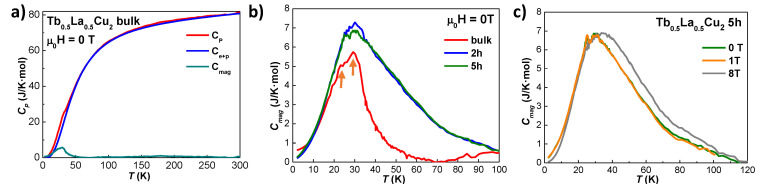
(**a**) experimental data for the specific heat c${}_{p}$ vs. *T* for Tb${}_{0.5}$La${}_{0.5}$Cu${}_{2}$ bulk alloy measured at $\mu_{0}H$ = 0 T (red line), together with the c${}_{e+p}$ (blue) and $c_{mag}$ (green) contributions; (**b**) magnetic specific heat ($c_{mag}$) vs. temperature (T) measured at $\mu_{0}H$ = 0 T for the bulk alloy (red line), 2 h milled (blue line) and 5 h milled (green line) NPs; (**c**) field dependency of the $c_{mag}$ vs. *T* for 5 h milled NPs measured at $\mu_{0}H$ = 0 T (red line), $\mu_{0}H$ = 1 T (green line) and $\mu_{0}H$ = 8 T (gray line).

The phononic $c_{ph}$ has been assumed to follow the Debye model and the electronic $c_{e}$ is considered linearly dependent with the temperature. Both contributions have been added together in one single term, labelled as $c_{e+ph}$, and subtracted from the total $c_{p}$ to obtain the $c_{mag}$. Values for the Debye temperature $\theta_{D}$ and the Sommerfeld coefficient γ have been taken from the non-magnetic isomorphous YCu${}_{2}$ [7], as it is a common practice [5,54,55]. A renormalization factor $\frac{\theta_{D}^{magnetic}}{\theta_{D}^{YCu_{2}}}$ has been applied to take into account the different molar masses between the R${}^{3+}$ ions and the Y${}^{3+}$ ones, in the same way as indicated in [56]. These contributions are shown in Figure 8a and Figure 9a.

Figure 8b shows the field dependence of the $c_{mag}$ for Tb${}_{0.5}$Gd${}_{0.5}$Cu${}_{2}$ bulk alloy. First, for $\mu_{0}H$ = 0 T, a clear peak, in the form of a λ-anomaly, shows at $T_{N}$ = 45.4(1) K. This peak is associated with the second order Néel transition. Its intensity decreases and shifts to lower temperatures when increasing the $\mu_{0}H$, as expected for AF transitions [57,58]. Second, below $T_{N}$, a huge broadening can be noticed between T∼ 20 K and T∼ 35 K. This shoulder is mainly triggered by the spin waves that propagate within the ordered magnetic moments, and constitutes a typical hint of an amplitude-modulated (AM) magnetic structure. Given that both GdCu${}_{2}$ [59] and TbCu${}_{2}$ [16] display this AM–AF structure, it would not be surprising that a dilution containing both ions will arrange in an AM structure as well. It is also possible that crystalline-electric-field effects could also contribute to this broad hump, as it has been observed in RCu${}_{2}$ alloys [60]. Third, for $\mu_{0}H$ = 3 T, it is worth mentioning the appearance of two additional peaks located at *T* = 34.3(1) K and T= 45(1) K (dark orange arrows). Whereas the narrow shape for former may be indicative of a first order transition associated with the existence of Ferromagnetic (FM) interactions (see $M_{DC}\left(H,T\right)$ measurements), the shape of the latter may be indicative of a second order Néel transition. Considering the magnetic characterisation, it is plausible that, when the external applied field is strong enough, the magnetic response of the Tb${}^{3+}$ and Gd${}^{3+}$ ions would be somehow decoupled, leading to two AF transitions that leave a double peak structure in the $c_{mag}$. In Figure 8c, a comparison between the bulk and the NP (t= 2 h) state is shown. It can be seen there how the intensity of the λ-anomaly from the bulk is reduced in the NP state. As for the bulk, the NPs show a broad hump located at around 25 K, mostly triggered by spin waves. Hence, this finding would reveal that the magnetic order survives within the nanoscale, but weakened with respect to the bulk situation. It is possible that this magnetic order would be triggered by the RKKY interactions involving Tb${}^{3+}$ ions, as the ones coming from Gd${}^{3+}$ have shown in [15] to not be strong enough to give rise to a magnetic collective order state at this NP size.

Figure 9b shows the evolution of the $c_{mag}$ starting from bulk Tb${}_{0.5}$La${}_{0.5}$Cu${}_{2}$ alloy to 5 h milled (〈D〉≈ 9 nm) NPs. The results for the bulk alloy are coherent with the magnetic characterisation measurements, as two peaks located at $T_{N}$ = 29.4(1) K and $T_{f}$ = 23.0(1) K (marked with dark orange arrows) are observed. These two peaks survive in the NPs at $T_{N}$ = 29.7(1) K and $T_{f}$ = 24.9(1) K, which further supports the already stated SAF state of the NPs. As it has been shown previously in the magnetic characterisation, these $T_{N}$ and $T_{f}$ get closer when reducing the size, together with an increase (decrease) in the magnetic signal associated with the SG (AF) state. These facts lead to a broadening of the peak (with a maximum value of ∼7 J/K mol), which should be associated mainly with the SG state. Figure 9c shows the $c_{mag}$ for Tb${}_{0.5}$La${}_{0.5}$Cu${}_{2}$ 5 h milled NPs, as the SG phase showed the most robust at that NP size for the series of Tb${}_{0.5}$La${}_{0.5}$Cu${}_{2}$ alloy. It can be noticed that the $c_{mag}$ is mostly field-independent at lower fields, whereas a shift towards higher temperatures happens for $\mu_{0}H$ = 8 T.

## 4. Conclusions

The investigation of three series of TbCu${}_{2}$ magnetic NPs ensembles diluted with magnetic and non-magnetic R${}^{3+}$ ions has been performed. We have proved that it is possible to obtain magnetic nanoparticles with substituted R-ions via high-energy ball milling. The Rietveld refinements of the XRD patterns reveal that the crystalline orthorhombic Imma structure of bulk TbCu${}_{2}$ parent alloy is retained in the diluted alloys, for both bulk and NPs. Furthermore, the microscopic analysis of the temperature dependent neutron diffraction patterns discloses the magnetic structure of Tb${}_{0.5}$La${}_{0.5}$Cu${}_{2}$ alloy (bulk and NPs). These follow the collinear commensurate structure evidenced by TbCu${}_{2}$ for bulk and NP states [16].

The selection of different *diluting* ions provides a very rich scenario with respect to the degrees of magnetic disorder that can be promoted in collections of magnetic NPs. Starting from the less disordered state, Tb${}_{0.5}$La${}_{0.5}$Cu${}_{2}$ NPs showed a SAF arrangement in which the AF order is retained within the NP core and the disordered SG phase is located at the shell. Then, progressing to a more disordered state, magnetic NPs of Tb${}_{0.5}$Gd${}_{0.5}$Cu${}_{2}$ revealed themselves to be a SSG ensemble, where all the magnetic moments have fallen into a frustrated state. In addition, finally, Tb${}_{0.1}$Y${}_{0.9}$Cu${}_{2}$ NPs could be placed at the most disordered extreme. Here, the AF order is absent already at the bulk state, where a CSG showed. The evolution with the size reduction leads to a gradual *dilution* of the interactions among the magnetic 4-*f*-moments, resulting in a weakly interacting SPM state for 〈D〉≈ 7.5 nm sized NPs. In summary, this work is a base from which to understand how the different degrees of magnetic disorder can be achieved by tuning the strength of the RKKY interactions in stable nanocrystalline metallic structures. These results are especially interesting in different research fields such as skyrmions and magnetocalorics.

## Figures and Tables

**Table 1:** Orthorhombic mean lattice parameters (*a*, *b* and *c*); unit size cell volume **V**, mean NP diameter 〈D〉, microstrain η, and Bragg factor $R_{B}$ for the produced diluted alloys.

Alloy	t(h)	a (Å)	b (Å)	c (Å)	V(nm)	〈D〉 (nm)	η (%)	$R_{B}$ (%)
Tb${}_{0.5}$Gd${}_{0.5}$Cu${}_{2}$	**bulk**	4.312(2)	6.858(5)	7.325(5)	216.6(3)	—	—	13.3
	**2 h**	4.319(3)	6.842(4)	7.313(4)	216.1(2)	9.0(8)	0.5(1)	6.6
	**5 h**	4.320(5)	6.839(6)	7.312(7)	216.0(1)	7.0(9)	0.6(1)	5.8
Tb${}_{0.5}$La${}_{0.5}$Cu${}_{2}$	**bulk**	4.381(5)	7.057(1)	7.416(1)	229.3(3)	—	—	24.5
	**2 h**	4.400(2)	7.084(4)	7.429(5)	231.6(2)	12.9(8)	0.4(1)	9.7
	**5 h**	4.421(5)	7.116(6)	7.478(8)	235.6(2)	9.0(9)	0.4(1)	8.7
Tb${}_{0.1}$Y${}_{0.9}$Cu${}_{2}$	**bulk**	4.302(4)	6.865(2)	7.295(2)	215.4(1)	—	—	16.2
	**2 h**	4.314(3)	6.878(2)	7.304(1)	216.7(1)	9.0(8)	0.47(9)	3.2
	**5 h**	4.310(4)	6.887(2)	7.317(3)	217.2(1)	7.5(4)	0.95(2)	1.7

**Table 2:** Néel temperature ($T_{N}$), freezing transition ($T_{f}$), paramagnetic Curie temperature ($\theta_{P}$) and effective magnetic moment ($\mu_{eff}$) obtained from Curie–Weiss fitting of FC measurements taken at $\mu_{0}H$ = 100 mT for the different produced alloys. The asterisk * indicates the blocking $T_{B}$ instead of $T_{f}$.

Alloy	t(h)	$T_{N}$ (K)	$T_{f}$ (K)	$\theta_{P}$ (K)	$\mu_{\mathrm{eff}}\left(\frac{\mu_{B}}{\mathrm{at}}\right)$
Tb${}_{0.5}$Gd${}_{0.5}$Cu${}_{2}$	bulk	47.2(1)	absent	19.9 (5)	9.26(1)
	2 h	absent	19.7(1)	16.1(1)	9.31(3)
	5 h	absent	21.2(1)	13.2(3)	9.86(1)
Tb${}_{0.5}$La${}_{0.5}$Cu${}_{2}$	bulk	33.1(1)	absent	20.3(2)	10.23(2)
	2 h	27.1(1)	6.2(1)	10.2(7)	10.16(2)
	5 h	26.3(1)	7.0(1)	7.3(1)	10.29(4)
Tb${}_{0.1}$Y${}_{0.9}$Cu${}_{2}$	bulk	absent	4.1(1)	2.84(1)	10.53(2)
	2 h	absent	3.5(1)	−0.34(4)	10.56(2)
	5 h	absent	3.0(1) *	−0.79(4)	10.76(6)

**Table 3:** δ-shift parameter, relaxation time $\tau_{0}$ of individual particles for f→0, freezing transition temperature $T_{f}$ and critical exponent zν for the diluted alloys. The fitting of the experimental data for 5 h-milled Tb${}_{0.1}$Y${}_{0.9}$Cu${}_{2}$ NPs didn’t converge to a critical slowing down, as the NPs are arranged forming an interacting-SPM ensemble.

Alloy	t(h)	δ	$\tau_{0}$ (s)	zν	T${}_{f,0}$ (K)
Tb${}_{0.5}$Gd${}_{0.5}$Cu${}_{2}$	2 h	0.058(2)	10${}^{-8}$	9.11(9)	18.7(5)
	5 h	0.049(2)	10${}^{-8}$	5.92(11)	21.51(7)
Tb${}_{0.5}$La${}_{0.5}$Cu${}_{2}$	2 h	0.070(4)	5 × 10${}^{-8}$	5.6(5)	7.4(1)
	5 h	0.077(3)	5 × 10${}^{-8}$	5.4(2)	7.5(1)
Tb${}_{0.1}$Y${}_{0.9}$Cu${}_{2}$	bulk	0.048(2)	10${}^{-8}$	6.66(14)	4.00(2)
	2 h	0.075(3)	10${}^{-8}$	6.5(4)	3.80(5)
	5 h	0.092(8)	——	—–	—–

## Supplementary Materials

The following are available online at https://www.mdpi.com/2079-4991/10/11/2148/s1, Figure S1: ZFC-FC magnetisation values normalised by the applied field, H = 1 kOe (M/H) vs. Temperature, T. All the insets show the Curie-Weiss fittings. Bulk alloys are represented in Figure S1a–c, 2 h milled ones, in e–g and 5 h milled ones, in i–k. In all of the cases, Tb${}_{0.5}$Gd${}_{0.5}$Cu${}_{2}$ measurements are presented first, Tb${}_{0.5}$La${}_{0.5}$Cu${}_{2}$, second and Tb${}_{0.1}$Y${}_{0.9}$Cu${}_{2}$ at the third place. In d,h,l, a linear fitting H${}^{2/3}$ vs. T has been employed to show that only bulk Tb${}_{0.1}$Y${}_{0.9}$Cu${}_{2}$ alloy follows a de Almeida-Thouless line. Figure S2:Magnetisation value (M) per R${}^{3+}$ ion, measured at $\mu_{0}$H = 8 T (6 T for Tb${}_{0.1}$Y${}_{0.9}$Cu${}_{2}$) vs NP mean diameter size, 〈D〉.

## Abbreviations

The following abbreviations are used in this manuscript:

NP	Nanoparticle
MNPs	Magnetic Nanoparticles
R	Rare Earth
t	Milling time
CEF	Crystal Electric Field
SG	Spin Glass
SSG	Super Spin Glass
CSG	Cluster Spin Glass
FM	Ferromagnetic
AF	Antiferromagnetic
SAF	Superantiferromagnetism
XRD	X-Ray Diffraction
ND	Neutron Diffraction
TEM	Transmission Electron Microscopy
Bragg Error Factor	$R_{B}$
$\mu_{0}H_{C}$	Coercitive Field
ZFC	Zero Field Cooling
FC	Field Cooling
