# Supplementary material for: Exploring the Different Degrees of Magnetic Disorder in TbxR1−xCu2 Nanoparticle Alloys
Source: Nanomaterials (Basel). 2020 Oct 28;10(11):2148. doi: 10.3390/nano10112148 (PMC7694043; doi:10.3390/nano10112148)
Supplement: Supplementary file 1 [file nanomaterials-10-02148-s001.pdf]

Supplementary Materials: Exploring the Different Degrees of Magnetic Disorder in $Tb_xR_{1-x}Cu_2$ Nanoparticle Alloys

Elizabeth M. Jefremovas [1,*], María de la Fuente Rodríguez [1], Javier Alonso [1], Jesús Rodríguez Fernández [1], José Ignacio Espeso [1], Inés Puente-Orench [2,3], Daniel P. Rojas [4], Ana García-Prieto [5], María Luisa Fdez-Gubieda [6], Lidia Rodríguez Fernández [7] and Luis Fernández Barquín [1]

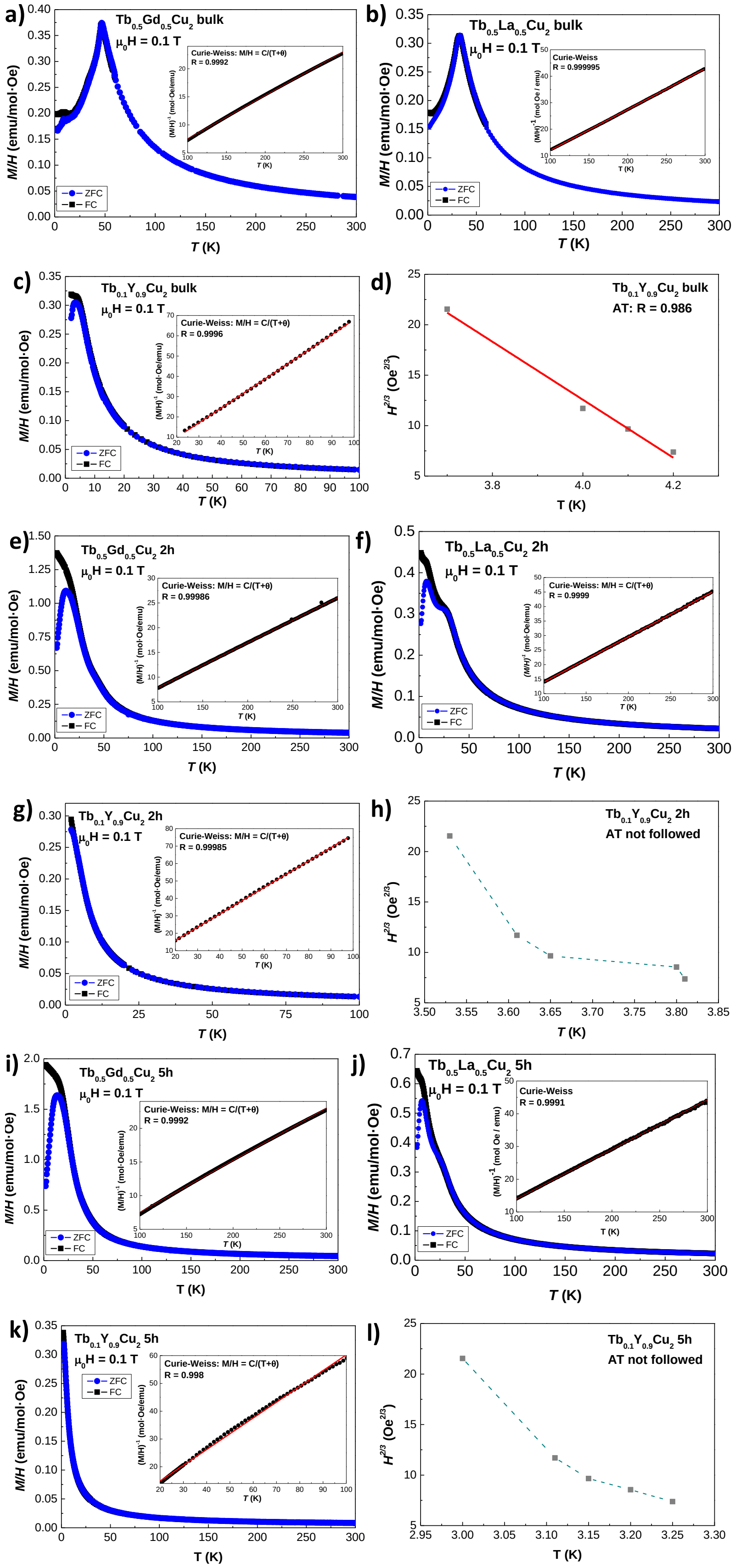

Figure S1. ZFC-FC magnetisation values normalised by the applied field, H = 1 kOe (M/H) vs. Temperature, T. All the insets show the Curie-Weiss fittings. Bulk alloys are represented in Figure S1 **a)** to **c)**, 2h milled ones, in **e)** to **g)** and 5h milled ones, in **i)** to **k)**. In all of the cases, $Tb_{0.5}Gd_{0.5}Cu_2$ measurements are presented first, $Tb_{0.5}La_{0.5}Cu_2$, second and $Tb_{0.1}Y_{0.9}Cu_2$ at the third place. In **d), h)** and **l)**, a linear fitting $H^{2/3}$ vs. T has been employed to show that only bulk $Tb_{0.1}Y_{0.9}Cu_2$ alloy follows a de Almeida-Thouless line.

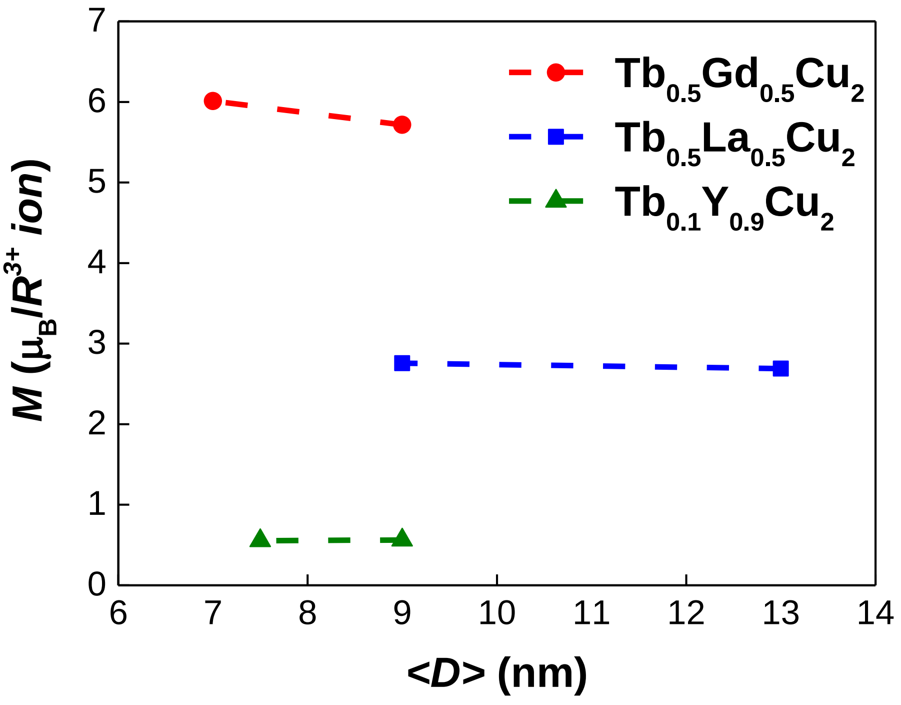

Figure S2. Magnetisation value (M) per R^{3+} ion, measured at $\mu_0 H = 8$ T (6 T for $Tb_{0.1}Y_{0.9}Cu_2$) vs NP mean diameter size, $\langle D \rangle$.
